# [Retracted] Knockdown of MALAT1 inhibits osteosarcoma progression via regulating the miR-34a/cyclin D1 axis

**DOI:** 10.3892/ijo.2026.5862

**Published:** 2026-02-24

**Authors:** Guangchao Duan, Chuanlin Zhang, Changke Xu, Chao Xu, Lei Zhang, Yan Zhang

Int J Oncol 54: 17-28, 2019; DOI: 10.3892/ijo.2018.4600

Following the publication of this paper, it was drawn to the Editor's attention by a concerned reader that certain of the β-actin control bands shown in Fig. 6D on p. 25 had already been submitted for publication to a different journal in an article written by different authors at different research institutes. In addition, it was also identified that invasion assay data in Fig. 4D and western blot data in Fig. 5E also subsequently appeared in articles in other journals that were written by different authors at different research institutes.

Given that the abovementioned contentious data in Fig. 6D had already been submitted for publication elsewhere prior to the submission of this paper to *International Journal of Oncology*, the Editor has decided that this paper should be retracted from the Journal. The authors were asked for an explanation to account for these concerns, but the Editorial Office did not receive a reply. The Editor apologizes to the readership for any inconvenience caused.

